# A Comprehensive Review of Computational Methods for Automatic Prediction of Schizophrenia With Insight Into Indigenous Populations

**DOI:** 10.3389/fpsyt.2019.00659

**Published:** 2019-09-12

**Authors:** Randall Ratana, Hamid Sharifzadeh, Jamuna Krishnan, Shaoning Pang

**Affiliations:** ^1^School of Computing, Unitec Institute of Technology, Auckland, New Zealand; ^2^Bay of Plenty District Health Board, Whakatane, New Zealand

**Keywords:** psychosis, schizophrenia, computational methods, language dysfunction, culture

## Abstract

Psychiatrists rely on language and speech behavior as one of the main clues in psychiatric diagnosis. Descriptive psychopathology and phenomenology form the basis of a common language used by psychiatrists to describe abnormal mental states. This conventional technique of clinical observation informed early studies on disturbances of thought form, speech, and language observed in psychosis and schizophrenia. These findings resulted in language models that were used as tools in psychosis research that concerned itself with the links between formal thought disorder and language disturbances observed in schizophrenia. The end result was the development of clinical rating scales measuring severity of disturbances in speech, language, and thought form. However, these linguistic measures do not fully capture the richness of human discourse and are time-consuming and subjective when measured against psychometric rating scales. These linguistic measures have not considered the influence of culture on psychopathology. With recent advances in computational sciences, we have seen a re-emergence of novel research using computing methods to analyze free speech for improving prediction and diagnosis of psychosis. Current studies on automated speech analysis examining for semantic incoherence are carried out based on natural language processing and acoustic analysis, which, in some studies, have been combined with machine learning approaches for classification and prediction purposes.

## Introduction

Psychosis is defined as a clinical syndrome composed of core symptoms of delusions, hallucinations, and thought disorder. The term psychosis was first coined in 1845. This concept then reflected a disorder of the interaction between the mind and a brain. In the late 19th century, Wernicke deconstructed psychosis into disorders affecting consciousness of body, outside of one’s personality and of the outside world. This sub-categorization was the first indication that the term psychosis was non-unitary. In the early 20th century, based on a medical model, Kraepelin defined psychosis as dementia praecox and manic depressive insanity. He considered dementia praecox as a neurodegenerative disease (today known as schizophrenia). In contrast to Kraepelin, Blueler renamed dementia praecox as schizophrenia. He was first to describe positive and negative symptoms. The term schizophrenia means split mind; fragmented thinking of people with the disorder (1, 2).

Shifting away from a medical model, based on a phenomenological approach to understanding psyche, Jasper hypothesized the layers of mental disorder as exogenous and endogenous. Psychosis according to Jasper was present in the three different levels of mental disorder: neurosis, exogenous, and endogenous. This definition was broadened to include hallucinations and delusions, which were the basis for loss of reality. This meant the term psychosis was present in all severe mental disorders and the core symptoms were hallucinations, delusions, and disordered thinking. Broad conception of psychosis led to Schneider introducing first rank symptoms as this form of categorization emphasizes some psychotic symptoms as more important than others (1, 2).

In terms of the current classification system of mental disorder in DSM-V and ICD-10, psychosis is defined based on clinically observable features and clinical picture. Thus, psychosis is a clinical syndrome composed of various symptoms. The degree to which the symptoms affect everyday function should not be part of the definition of psychosis. The presence of necessary symptoms should suffice to diagnose psychosis on a level of clinical observation.

In terms of current classification system, the core symptoms of schizophrenia are delusions, hallucinations, disorganized speech, and behavior and other symptoms that cause social and occupational dysfunction. Symptoms must be present for 6 months and include at least 1 month of active symptoms for a diagnosis. The current DSM-V has raised the symptom threshold requiring that the individual have two of the specified symptoms. The diagnostic criterion has moved away from subtypes. Instead, some of these subtypes are specifiers that help provide further details to the diagnosis. These specifiers can be used in other disorders such as bipolar and major depression.

The definitions of psychosis and schizophrenia have been recently considered by the diagnostic classification system as needing to include a cultural formulation. However, the cultural definition of psychosis and schizophrenia has different meanings and outcomes in various cultures. The current definition of psychosis and schizophrenia has been challenged by the biological and psychological interpretation and addresses issues such as colonization, indigenous worldviews, and spirituality.

Hence, the construct of schizophrenia is heterogeneous. It is also disjunctive. This means that one person receiving the diagnosis has nothing in common with another person with the same diagnosis (3). The poor reliability and validity of the concept of schizophrenia has led to many researchers focusing on discrete psychotic phenomenon such as hallucinations, delusions, and language abnormalities. One study by Soares-Weiser (4) found that first rank symptoms of schizophrenia (FRS) correctly identified patients with schizophrenia 75% to 95% of the time. The use of FRS to diagnose schizophrenia in triage will incorrectly diagnose around 5 to 19 people in every 100 who have FRS as having schizophrenia. This study concluded that people identified with schizophrenia based on FRS will still merit specialist assessment and help due to the severity of disturbance in their behavior and mental state (4).

The different conceptions, assumptions, and formalism originating out of medial model and phenomenology and descriptive psychopathology meant that there was a need to quantify symptoms of mental disorder.

Clinical assessments in psychosis are based on clinician-rated and patient self-report form. In assessing psychotic symptoms, BPRS is the most widely used clinician-rated instrument. This is a scale designed to measure several psychiatric symptoms: mood, behavioral, and psychotic symptoms. The Positive and Negative Symptom Scale (PANSS) is a scale designed to assess severity of psychotic symptoms and encompasses scales of positive and negative symptoms and general psychopathology. Signs and Symptoms of Psychotic Illness is a recent rating scale used to measure psychopathological processes common in psychosis. This rating scale was designed to overcome the limitations of BPRS (Brief Psychiatric Rating Scale) and PANSS (5).

There are other scales used for research context-specific interviews and symptom-based instruments such as Diagnostic Interview for Genetic Studies, Diagnostic Interview for Psychoses, and Psychiatric Interview for Genetic Studies, as well as symptom specific ones such as Clinical Assessment Interview for Negative Symptoms and Psychotic Symptom Rating Scale for hallucinations and delusions. In conclusion, symptom-specific instruments were practical for clinical contexts and comprehensive in terms of symptom severity, but the diagnostic valence was not sufficiently addressed (5).

PANSS rating scales remain the primary mode for assessing and diagnosing schizophrenia by clinicians and researchers. This scale is used to monitor severity of positive and negative symptoms and track treatment response. There are fewer articles on the utility of newer scales like CAINS (Clinical Assessment Interview for Negative Symptoms) and the BNSS (Brief Negative Symptom Scale) compared to older scales such as PANSS, SANS (Scale for the Assessment of Negative Symptoms), NSA-16 (Negative Symptom Assessment-16), and CGI-SCH (Clinical Global Impression Schizophrenia) (5).

Screening for psychosis using existing rating instrument such as structured interview for psychosis risk syndromes (SIPS) and clinical high risk (CHR) criteria has its own set of problems such as its use in diverse samples with the goal of validating assessments, screening populations for clinical referral, recruiting samples of interest for research participation, and estimating symptom prevalence and severity (6). One such study that reflects the problems with the concept of CHR by Mamaha was the overestimation of psychosis prevalence rates in Kenyan youth considered to be clinically high risk for psychosis. This study concluded that assessment tools such as psychosis risk screening instruments were not cross-culturally applicable (7).

Given the diverse use of screening tools, reliable tools are needed to establish reliable norms and screening thresholds as score elevations are unreliable markers of high-risk psychosis across populations and settings (6).

Other than the main limitation of culture, the quality and general utility of each scale vary. Its quality is determined by the validity and reliability of the scales. The utility of the scale is determined by the time of administration and the setting for which the scales can be administered in research or clinical settings and can present as with high inter-rater reliability (8). Older scales compared to current scales do not incorporate negative symptoms. CAINS and BNSS are considered reliable due to their concise and accessible format. To conclude, there is currently no scale that is simple and user friendly that incorporates a multidimensional model of schizophrenia that addresses psychosocial and cognitive components that would be consistent with a recovery model in mental health.

Thus, expert clinicians have to rely on clinical observation and base their diagnosis on behavioral presentation, speech, and language. Expert clinicians and psychiatrists are not free from biases (confirmation bias, cultural and racial bias) and are more likely to give a diagnosis of schizophrenia as it was perceived to benefit the patient in terms of accessing health care resources. On the other hand, other lay clinicians including psychologists are less likely to make and give a diagnosis of schizophrenia due to differing perceptions of the condition. Furthermore, most clinicians struggle with using the current diagnostic classification of schizophrenia that does not lend itself easily specifically in recognizing its presence in its early stages of illness formation (9).

The definitions of psychosis and schizophrenia have been recently considered by the diagnostic classification system (DSM-V) as needing to include cultural formulation. However, the cultural definition of psychosis and schizophrenia has different meanings and outcomes in various cultures. The current definition of psychosis and schizophrenia has been challenged by the biological and psychological interpretation and addresses issues such as colonization, institutional racism, politics, indigenous worldviews, and spirituality. It is important to know how indigenous cultures perceive, define, and respond to mental disorders in their own perceptual and conceptual terms. Without such phenomenological understanding, it is clear that Western classification systems of psychiatric diagnosis have distorted the cultural and social reality of non-Western populations. Psychosis is not defined solely in terms of socially disruptive conduct but instead is a process whereby the person that becomes socially recognized as psychotic is a complex negotiation with moral and legal implications. In conclusion, it is imperative that early intervention psychosis services reflect the context in which it has been set up and implemented and rolled out that is the service meets the diverse needs of its communities and addresses the geographic, cultural, and political realities and it starts with acknowledgement that psychosis and schizophrenia for indigenous and non-Western cultures has a “classification system of its own” compared to existing psychiatric diagnostic classification systems and does not necessarily reflect pathology but may be measures of degrees of separation of wellness and health.

This paper aims to review existing and current studies on language anomalies in schizophrenia and then examine existing and current studies on this topic from the point of view of non-Western and indigenous cultures. Then, by moving away from linguistic studies, this paper aims to extensively review past and current literature on computational methods of analysis and prediction of psychosis and other major mental disorders. Finally, this paper will review the state-of-the-art methods based on machine learning approaches for automatic classification of psychosis and schizophrenia within a natural language processing (NLP) framework.

## Schizophrenia and Language Abnormalities

Psychosis is a symptom characterized by hallucinations and delusions and includes disturbance in speech and language. Schizophrenia, on the other hand, is characterized by disturbances in speech, thinking, motivation, and volition.

The similarity between schizophrenia and psychosis is that most individuals exhibit abnormalities in language such that it is considered a major symptom for diagnosis. Compared to psychosis, language disorder in schizophrenia has been clinically characterized in detail in many studies.

There is a wide range of literature on language anomalies in schizophrenia. A study by Covington derived from a survey on schizophrenic language summarized key findings that included phonetics, morphology, syntax, semantics, and pragmatics (10). This study identified two types of impairment: thought disorder (unable to follow a discourse plan) and schizophasia (unintelligible utterances). Thought disorder was described as a disruption of syntax–semantics interface. Schizophasia was thought to be a disruption at another level. Phonetics in individuals with schizophrenia was often abnormal. However, this study found that phonological structure, morphology, and syntax were normal or near normal (10). Access to lexicon was clearly impaired and characterized by stilting of speech and word approximation and neologisms.

Language disturbances in schizophrenia can be categorized as language output and language comprehension (11). The first approach was based on the statistical properties of language. In a given sentence, the predictability of individual words in a sentence of discourse text was measured using a type/token ratio. Type is defined as the number of different words in relation to the total number of words (token). A technique called Cloze Analysis (12) was used to measure predictability and type/token ratio was used to measure flexibility and variability in words. Based on studies on findings from severely thought disordered patients, a score was generated (12). The clinical rating scale for thought disorder originated from this approach to measuring severity of disturbances in thought form but also gave rise to the assumption that speech and language disturbances were associated with disturbances in the form of thinking (12).

The second approach was based on lexical and syntactical structure pioneered by Chaika (13, 14). In this approach based on classical clinical observations, each patient’s speech was carefully documented and described. Chaika also proposed that sentences were produced according to semantic features of previously uttered words rather than based on a topic, thereby producing lexical and syntactic errors. When compared to healthy individuals, these findings in a patient with schizophrenia were considered to be in an exaggerated form (14, 15). Another study by Hoffman and Sledge (16) found that the speech disturbances in patients with schizophrenia were “more grammatically deviant” and “less syntactical complex” compared to controls. However, this specific finding was felt to be linked with earlier onset of illness, longer duration of illness, and negative symptoms.

A third approach to language output was focused on discourse structure (17–19). The focus was on the use of cohesion devices that lined words with real words referent and with previous referents. This approach leads to another tool of measurement called Communication Disturbances Index (20). This measurement tool sub-divided the patient’s referential impairments into categories such as vague, missing information and confused reference (20). **Table 2** summarises the linguistic models in schizophrenia. Other forms of abnormalities in language structure and form have been summarized in **Table 1** covering other studies (15, 21–24) with respect to the type of language abnormalities they studied. As it is evident in **Table 1**, language disturbances can vary from a semantic to syntactical to discourse level to severe forms of language disturbances manifesting as thought disorder and expression of meaning.

**Table 1:** Table of language abnormalities.

Study Authors	Language Abnormalities
Rochester, Martin (15)	Thought disorder, patient discourse
Kuperberg, Caplan (21)	Thought disorder, speech disorder
Rochester and Martin (15)	Phonology, morphology, semantics, pragmatics
Goldberg (22)	Thought disorder separate from speech disorder
Andreasen (23)	Illogical thinking, incoherence
Flack et al. (24)	Disordered processes, discrepancies in expression and feeling

**Table 2:** Table of linguistic models.

Study Author	Linguistic models
Rochester and Martin (15)	Cohesion analysis
Andreasen (23)	Syntactical analysis
Maher (12)	Textual analysis of discourse, cloze procedures, type/token

Language comprehension in patients with schizophrenia is subtle compared to language production (output) and is less well documented. Disturbances in language comprehension is characterized by patients’ difficulties with figurative language. Some patients present with concrete thinking. Abstract thinking through the use of proverbs and metaphors was commonly used to assess thought disturbances in schizophrenia. This has been demonstrated in various studies that confirmed a common finding that patients with schizophrenia preferred concrete interpretations when asked to interpret figurative language. Newer studies have proposed new ways of conceptualizing language dysfunction such as propositional and deictic meaning (25, 26).

According to DSM-V criteria, a diagnosis of schizophrenia requires at least one of the three positive symptoms (hallucinations, delusions, and disorganized speech). None of these symptoms were inherently related to language disturbances.

DSM-V describes formal thought disorder as disorganized thinking and is inferred from an individual’s speech. The speech patterns range from patients who are productive and communicate well, or speech is produced and the listeners struggle to make sense of the content, or in a classical profile, the different forms of thought disorder characterized by loss of goal, derailment, and tangentiality.

Rosselló et al. described a linguistic model that described how positive symptoms were inherently a form of language dysfunction. In this paper, the authors proposed a linguistic model underpinning the three positive symptoms of hallucinations, delusions, and disorganized speech. The linguistic model describes the one-to-one correlation between human specific thought or meaning and forms of grammatical organization as an integrative and co-dependent view of linguistic cognition and its sensory-motor dimensions. The authors further explain the core dimensions of meaning-mediated grammar as forms of referential and propositional meaning. Hence, based on this model, the positive symptoms of schizophrenia is a failure in language-mediated form of meaning manifesting as either a disorder of speech perception (auditory verbal hallucinations), abnormal speech production running without feedback control (formal thought disorder), or production of abnormal linguistic content (delusions) (27).

Thus, it is important to understand the relationship between specific cognitive abnormalities and the clinical symptoms of schizophrenia. Andreasen et al. generated composite scores for negative, disorganized, and psychotic symptom ratings in 134 patients with schizophrenia (based on DSM-IV criteria). Partial correlations were computed with neuropsychological measures. This study found that negative symptoms were related to poor performances on tests of verbal learning and memory, verbal fluency, visual memory, and visuo-motor sequencing. Disorganized symptoms (positive FTD, inappropriate affect, and bizarre behavior) were correlated with lower verbal IQ and poor concept attainment. Psychotic symptoms (delusions and hallucinations) had no significant relationship with cognitive deficit (28).

Older studies supported Andreasen’s hypotheses that negative and positive symptoms (disorganized) are associated with cognitive impairment. The pattern of cognitive deficits associated with negative symptom is different to disorganized symptoms (29). This further suggested that these two symptom dimensions had different neurobiological substrates. This study by Andreasen is further supported by earlier studies that found that paranoid patients with good pre-morbid adjustment had shorter duration of illness and showed little or no cognitive impairment. The paranoid subtype described in these studies is of high-level delusions and may represent a group of schizophrenic patients in whom there is no relationship between psychotic symptoms and cognitive dysfunction (30).

Hence, DSM-III-R and DSM-IV explicitly incorporate this lack of association between psychotic symptoms and cognitive dysfunction into their diagnostic criteria for the paranoid type of schizophrenia. The diagnostic criteria for the paranoid type in DSM-III-R and DSM-IV include prominent delusions and the absence of cognitive dysfunction.

In conclusion, although it is easy to recognize schizophrenia speech to some extent, the same cannot be said about defining all of its features due to anomalies at multiple levels of language processing. By reviewing existing literature, we tried to classify some of these abnormalities associated with schizophrenia in this section. This review showed that in individuals with formal thought disorder, there were greater disturbances in language, and in persons with no formal thought disorder, there were least disturbances in language (speech volume and syntactical complexities). Schizophrenia is characterized by positive symptoms (hallucinations, delusions, disorganized thinking), negative symptoms (alogia, apathy, amotivation, and avolition), and cognitive dysfunction, which is not a diagnostic criteria. Schizophrenia affects cognitive abilities in the domains of attention, memory, processing speed, social cognition, and executive functioning. Cognitive dysfunction precedes, coincides, and outlasts positive symptoms. (31, 32).

In the next section, the focus is on the specific phenomenon of language disturbances and schizophrenia within a cross-cultural context.

## Cross-Cultural Findings of Language Disorder in Schizophrenia

Schizophrenia is found worldwide in diverse cultures. Within a Western model of schizophrenia, this condition has been purported to be fundamentally similar across all cultures with the only difference in its content and form (33). However, this supposed universality of the incidence and prevalence of schizophrenia has been challenged (33). The lifetime prevalence and incidence of this disorder varies significantly in time and place (33).

According to a review by Viswanath, delusions encountered in schizophrenia were found to be related to patient’s social, cultural, and social background (34). Religious delusions were common in Christian communities and rare in Hindu, Buddhist, and Muslim religion. Magical delusions were common in rural communities. The first large-scale cross-cultural evaluation of hallucinations found that visual hallucinations were more common in Africa. Another study found higher occurrence of auditory and visual hallucinations in non-European patients than in European patients. The ISPS showed that auditory hallucinations were commonest in all cultures and that visual hallucinations were the commonest in Africa and the rarest in Pakistan. This review concluded that FRS was culture free (34).

VK Varma (35) found that language and thought differences underpinned the main cross-cultural differences in symptoms and subtypes observed in schizophrenia. This study posited that greater linguistic competence leads to elaborate systematized delusion. This was associated with a poor prognosis. Low linguistic competence instead was considered as preventative of formation of elaborate delusion.

Another study of symptoms of schizophrenia in a community in Borneo (20) showed that cultural conception of thinking and feeling shaped individual experiences. Translation from English to Iban of present examination states such as thought insertion and withdrawal led to difficulties. Similarly, in another study examining the predictability of speech in Nigerian patients with psychosis using Cloze technique, it was found that the speeches were significantly less predictable. That said, the poor predictability of speeches of patients with psychosis showed significant correlations with presence of formal thought disorder. This study concluded that speech impairment observed in psychosis was universal across cultures, but the same could not be concluded about predictability of speech alone in the diagnosis of psychiatric patients (36). A study by Toppelberg found that language competence varied with psychosis and those in an acute psychotic state were often unable to express themselves in their second language (37).

In another study examining language content in Turkish patients in acute phase psychosis, using computer content analytic procedure, it was found that the speech content of Turkish patients with *schizophrenia* exhibited considerable similarity to that previously observed in American subjects, but there were certain dissimilarities that appeared to reflect the impact of culture on the manifestations of the schizophrenic disorder (38). The phenomenological differences between the three psychiatric syndromes compared were also reflected in the results of the content analysis. The most dissimilar syndromes were mania and depression, whereas the most similar were mania and *schizophrenia* (38).

Studies on bilingualism and psychosis for example found that people with schizophrenia who acquired a second or foreign language presented with linguistic deficits that were not as prominent (in some instances, altogether absent) when patients use their non-dominant language (39, 40, 90). This is further supported by another study in bilingual patients with psychosis that showed that more language abnormalities were found in the English language compared to a native language (41, 42). Some studies found that in multilingual patients with schizophrenia, one language may become less fluent and ungrammatical while other languages were semantically and grammatically coherent (43).

Maori (Indigenous population of New Zealand) understanding of extraordinary experiences and symptoms of schizophrenia often have a predominant explanation of being spiritual. In a study by Taitimu and Read (44), Maori participants were described as “tended to hold multiple explanatory models.” In this study, participants viewed experiences of psychosis as being due to a Maori ailment (Mate Maori), a gift (Matakite), or a Pakeha disease (Western psychiatric illness). However, the borders between these explanations varied by participant and depended on the experiences’ content, control, and context. The most common ways of understanding these experiences were spiritual and cultural in nature, although other explanations included psychosocial, biomedical, and historical trauma-related reasons (44).

Another study by Te Aonui and T. Kake (45) examined the cognitive neuro-psychological functioning in Maori diagnosed with schizophrenia. This study found that there was limited evidence on key clinical features of schizophrenia in Maori to inform decision making. Using clinical measures to assess a range of cognitive functions, this study found that Maori diagnosed with schizophrenia had greater impairment of verbal memory. The cognitive impairment was independent of psychotic symptoms but was associated with a higher anti-psychotic dose, higher anti-cholinergic load, and longer duration of illness (45).

There are currently no studies examining language disturbances in Maori patients in a bicultural setting. However, a study by Waters (46) might provide some insights into the link between deficits in verbal memory and language dysfunction (speech perception) observed in patients with schizophrenia. Waters (46) found that patients with schizophrenia showed difficulties with integrating speech content and speaker identity in memory, which was measured based on a gender-identity recognition task. The study found that female voices had more complex vocal characteristics and required greater integration compared to male voices. This study asserts that based on their findings, memory binding impairments may result in degraded or incomplete memory traces as the task requirement became increasingly complex (46). This study did not take into account the influence of culture. This study highlights the importance of considering cultural influences on verbal tasks and acquired knowledge (47).

A study (48) found that Mandarin-speaking patients showed impairments in basic auditory processing unlike Western groups, which relates to deficits in word recognition and social outcomes. Compared to the Western group, tonal deficits were related to emotional processing of speech but language processing was minimally affected. Language differences and tone were identified as an important variable in language disturbances in schizophrenia (48).

The only automated study in a cross-cultural setting of Singapore analyzing speech and language disturbances in schizophrenia at a lexical level found that the results of the study were limited by the accuracy of speech recognition as the accuracy of converting Singapore English to text was significantly lower than for native English (UK, US) (49).

In summary, these studies showed that a complex relationship exist between language and psychosis within cultural context. Old and current literature examining language disturbances in patients with schizophrenia in a cross-cultural setting have resulted in inconsistent findings. Cross-cultural similarities outweigh the differences in nature of presentation of schizophrenia and it is clear that cultural factors affect the course and pattern of its symptoms.

Although language manifests as a seamless whole with phonology, syntax, semantics, and pragmatics processes working together, underpinning this is the many dissociable mechanisms that underlie linguistic competence. It is difficult to conclude with certainty that positive symptoms come from verbal cognitive deficits. There is only one study that describes the relationship between language competence and delusions and catatonia. In addition, little attention has been directed in cross-cultural research to address the extent of cultural modulation of positive symptoms (cross-cultural construct that is robust).

In sum, it is imperative that researchers in natural language studying speech and language disturbances observed in schizophrenia understand the characteristics of speech and language features in a context of multiple world-views (44, 45).

## Computational Methods

Computational methods aim to model the abnormal patterns of the brain and attempt to relate it to normal function of the brain. There are two approaches to computational method: data analysis methods from machine learning (standard statistical methods) and theory-driven models that mathematically specify mechanistically interpretable relations between variables (observable variable, postulated and theoretically meaningful hidden variables) (50, 51). In the next section, we will review NLP and latent semantic analysis (LSA) techniques and current studies as to how it addresses specific symptoms of psychosis and schizophrenia. Using statistical methods, machine learning has demonstrated an ability to detect subtle features of psychosis in language. Semantic coherence, semantic density, and acoustic analysis are the current methods used in detecting early signs of psychosis. Machine learning can measure the linguistic variables, semantic coherence and semantic density, and use of words relating to sound.

### Natural Language Processing

NLP is a technology used to aid computers in understanding human’s natural language. NLP applies algorithm to identify and extract natural language rules. The result is unstructured data that are converted into an understandable form for computers. Syntactic analysis and semantic analysis are the main techniques used to complete NLP tasks. There are several techniques involved in syntactical analysis such as lemmatization, parsing, parts of speech tagging, word segmentation, sentence breaking, stemming, and morphological stemming.

Semantics analysis comprises application of computer algorithms aimed at understanding the meaning and interpretation of words and how sentences are structured. Techniques involved in semantic analysis are named entity recognition, word sense disambiguation, and natural language generation.

#### Latent Semantic Analysis

LSA is a technique in NLP, also known as a bag of words approach. It is a high-dimensional associative model that is based on the concept that word meaning is a function of the relationship of each word to every other word in a sentence (52). The LSA method used in current studies on automated prediction of psychosis enabled the development of predictor of transition of psychosis based on speech analysis. The predictors of speech dysfunction in patients at risk of developing schizophrenia were divided into speech pre-processing and speech analysis and cross-validation (53).

For the purpose of semantic analysis, LSA is trained on a corpus of transcriptions and collection of speech. Studies that used this method of cross-validation used specific corpus databases (53, 54). This method was used as LSA does not contain humanly constructed dictionaries, knowledge base, semantic networks, grammars, syntactic parses, or morphologies. LSA takes as it inputs raw text parsed into words and defined as unique character strings and separated into meaningful passages such as sentence and paragraphs. As such, this will result in many LSA models. These models will differ in terms of linguistic items: words, content-words, nouns–verbs, and main concepts. It is computationally challenging to produce models from large datasets such that it can provide a large coverage for the actual vocabulary in use. Hence, large LSA models are rare. In addition, the scores assigned by these models are strongly correlated with human judgment.

LSA theory is based on a linear associative model that embodies no human knowledge beyond its general learning mechanism to analyze a large corpus of natural text and generate a representation that capture the similarity of words and text passages. On that note, LSA is a distributional model and therefore is not an efficient representation of human language and communication. Representation based on LSA model is dense and cannot be indexed based on individual dimensions. Furthermore, the theoretical foundation of the LSA model to a large extent was considered as incomplete (52).

Thus, based on current studies that used the LSA method, the limitation of this language model lies in the gap in knowledge about what is normal across development for automated linguistic variables (53, 54). LSA does not handle all aspects of language processing but offers a biologically and psychologically plausible mechanistic explanation of the acquisition, induction, and representation of verbal meaning.

Evolving technology and intensive methodologies for cognitive studies in schizophrenia such as LSA have enabled further study of the differential cognitive deficits observed in schizophrenia. The wide-ranging ability offered by LSA based on its ability for cognitive modeling of learning and memory processes and for computing coherence in language and thought process has enabled a finer-grained method for evaluating cognitive dysfunction (verbal fluency task), memory processes (semantic and working memory), and discourse organization (thought disorder) compared to more subjective or observational measures of thought disorder.

LSA works by applying dimension reduction to local co-occurrence data from a large collection of documents after performing singular value decomposition on it. When the reduction is applied, the system forms condensed representations for the words that incorporate higher-order associations. The higher-order associations are primarily responsible for any semantic similarity between words in LSA. A memory model is described that creates semantic representations for words that are similar in form to those created by LSA. However, instead of applying dimension reduction, the model builds the representations by using a retrieval mechanism from a well-known account of episodic memory. Kuperberg in his study hypothesized that thought disorder reflects a deficit in semantic processing that can be dissociated from deficits in semantic and working memory. This study also suggested evidence that cognitive symptoms were independent of positive symptoms. The apparently contradictory local semantic coherence and higher-order structural incoherence (thought disorder) can be explained by the opposite tendencies between the semantic associative inclination on one hand and the incapacity to monitor higher-order structural planning on the other hand (55, 56). Hence, a clear advantage of LSA is its ability to demonstrate that negative symptoms and cognitive deficits are related but separable.

Positive symptoms and cognitive deficit are unrelated and can be discriminated. An example of the usefulness of NLP in treating delusions is described in a paper that postulates that delusions may be treated from delusional assertion into a non-delusional one, for example, by using a three-step linguistic therapy model. Syntactic priming, the tendency to reproduce specific language structures that one is exposed to, suggests that the therapist’s language use may be critical. This notion requires the traditional belief that language is simply a way of expressing thought to be challenged; it relies instead on a more nuanced understanding of a closer interdependence of thought and language.

In conclusion, the ultimate goal of LSA is data mapping, which provides information beyond the lexical level and reveals semantical relations between the entities of interest. Thus, because of its generality, LSA is a valuable analysis tool with a wide range of applications.

#### Parsing

Parsing refers to the formal analysis by a computer of a sentence of string of words into its constituents (57). By using current studies that have used parsing as part of NLP, the following discussion will focus on the different approaches to parsing and the limitations of this technique.

Modern parsing techniques are partly statistical and rely on a corpus of training data already annotated (parsed by hand). This approach allows the system to gather information about the frequency with which various constructions occur in specific contexts. Modern parsing approaches used include probabilistic context free grammars (PCFGS) (57), maximum entropy, and neural nets.

Parsing some forms of grammar formalism using computational methods is difficult. Thus, often some kind of context-free approximation to the grammar is used to perform first pass. Computational methods that use context-free grammars rely on the CYK (CockeYoungerKasami) algorithm, which is a rule-based parser (58). This algorithm is used to decide whether a given string belongs to a language of grammar or not. The CYK algorithm only operates on context-free grammar given in Chomsky Normal Form (CNF). Normal forms give more structure to work with, resulting in easier parsing algorithms.

Automated studies that used parsing as part of the NLP technique in assessing semantic cohesion used a generalized approach to parsing. This is a framework that uses both rule-based and statistical-based parsing algorithms. A generalized algorithm consists of five components, such as grammar, logic, semi ring, search strategy, and termination condition, that address problems with computational methods of parsing some forms of grammar (58).

A limitation of this approach to analysis was developing a qualitative parser for phonetically and morphologically rich languages. Literature is limited on different parsers developed for other languages that are required for NLP applications in machine learning; however, one example can be seen in (59) in which parsers are developed based on a method that suited free word order languages (e.g., Urdu).

Parsing is a technique in NLP that is useful in studying some groups of patients with schizophrenia who have intact lexical processing but impaired grammatical processing. One such study described this group of patients with schizophrenia as worse at producing the past tenses of regular and novel than irregular verbs, as compared to healthy control subjects. This pattern supports the dual system hypothesis that grammatical processing is impaired in schizophrenia, while lexical processing remains relatively spared, at least for forms like irregulars, which are presumably learned well before disease onset. Additionally, patients’ thought disorder scores predicted their performance at regular and novel (but not irregular) past-tense production, consistent with previous findings suggesting a relationship between thought disorder and grammar. This study demonstrates the ability offered by LSA to model neurocognitive deficits observed in language disturbances observed in patients in schizophrenia. In this study, the model is based on a single mechanism that posits that the learning and use of language depend on a single computational mechanism and therefore assume no *a priori* distinction between lexicon and grammar. One such model hypothesizes that the computation of irregular inflected forms depends particularly on semantics (in comparison to regular verbs), while inflection of novel forms depends particularly on phonology. Simulations of phonological damage to the model revealed a pattern of greater impairment on the production of past-tense forms for novel verbs than for regular and irregular verbs (which in turn were similarly impaired).

#### Speech Tagging

Part of speech tagging is the process of marking up a word in a text (corpus) as corresponding to a part of speech based on both its definition and its context (60). This process considers relationship with adjacent and related words in a phrase, sentence, or paragraph. The following section will discuss the various approaches to speech tagging and the important tasks it has in NLP and of its limitations.

In a rule-based tagging approach for example, software such as Part-of-Speech-tagger (POS tagger) reads texts in some language and assigns parts of speech to each word such as noun, verb, and adjective (52). A statistical-based tagger approach uses a cross-validated classifier based on machine learning algorithm in two stages: learning the underlying patterns using sub-set and the other predicting labels of samples not used during the learning stage (61). Lastly, a transformational based approach focuses on using other methods of cross-validation of semantic coherence.

Only one current study (53) has utilized speech samples analyzed using POS-Tag. This consisted of labeling every word by its grammatical function. Cross-validation was performed against classical literature aimed at testing for different levels of disorder within a range of texts modified according to semantic coherence. Ultimately, this method was aimed at testing whether the computational method could detect modifications. This saves new rules learned in the process for the future (53).

These techniques based on Western languages have shown a satisfying performance of 94%. However, other supervised POS-Tag technique in non-Western languages requires a large amount of annotated training corpus to tag properly (59). A limitation of this study (53) was the ability to develop an equivalent POS-Tag for the various non-Western languages as comparable with English POS-Tag. Non-Western languages do not have similar grammatical rules as the English language. Studies done in different languages require different POS-Tag models; for example, Southeast Asian languages tend to use stochastic tagging models (59).

Speech tagging based on the right context for a given language is important as some languages exhibit systematic ambiguities of words that can be correctly disambiguated only by the right context.

POS tagging is a fundamental step required by various NLP systems. It is an NLP technique that offers a method for developing sentence classification models for a range of severe mental illness (SMI) symptomatology. POS tagging is useful in terms of generating sentence classification given that the diagnostic semantics reflects common conditions in mental health based on classification taxonomies; it is the symptomatology of a condition that is used by clinicians to determine an appropriate treatment plan. This is due to the broad symptomatic manifestations of mental disorders, in the sense that, at a given time, a patient assigned a diagnosis (such as schizophrenia) can present with all, many, or very few of the symptoms associated with the condition. This is particularly pertinent to clinical practice where diagnoses are not necessarily assigned using research criteria. The problems of diagnostic semantics are especially apparent in SMI (schizophrenia, schizoaffective disorder, and bipolar disorder). This method allows for automatic extraction for many of the most informative symptoms from the patient narrative. Hence, the free text portion of the mental health EHR contains a potentially vast and complex tapestry of clinical information that, to date, has been effectively “invisible” when it comes to the generation of data specifically for clinical evaluation. The disadvantage, however, is that the annotation process is both knowledge-intensive and time-consuming in the clinical domain and the training of a POS tagger relies on sufficient quality annotations.

The advances in NLP due to enhanced computer processing power (Moore’s Law) have resulted in gradual lessening of use of Chomskyanth theories of linguistics. Chomskyanth theoretical underpinnings discouraged the use of corpus linguistics that underlies machine learning approach to language processing. For the purpose of this article, Chomsky theory states that all individuals are born with innate knowledge of grammar that serves as a basis for language acquisition. Everett in his study challenged Chomsky’s theory based on his findings of the language structure of the Pirahã people indigenous to Brazil. The language of the Pirahã people lacked grammatical constructs and several kinds of words commonly found in all languages. The absence was not due to inherent cognitive limitations but due to cultural values, i.e., immediacy of experience principle (62).

To conclude, the current trend in NLP research is focused on statistical models. These models have enabled probabilistic decisions based on attaching real-valued weights to the features that make up the input data.

Many speech recognition systems rely on statistical models that allow for more robust unfamiliar inputs, specifically one that contains errors (real-world data) and produces more reliable results when integrated into a larger system that comprises multiple subtasks. The use of POS tagging introduced the use of hidden Markov models to NLP. Hidden Markov model is defined as Markov chain for which the state is only partially observed. Observations are related to the state of the system but are typically insufficient to precisely determine the state. One common use is for speech recognition. The observed data is the speech audio waveform and the hidden state is the spoken text.

In the next part of this article, we will review studies that used automated speech analysis in patients with a psychotic illness and schizophrenia.

## Overview of Earlier Studies Using Automated Speech Analysis

Early studies in automated speech analysis were limited in the ability of its software to organize speech and language material. Thus, analysis of patient’s oral contributions was conducted at a discourse level. One such study conducted by Noel-Jorand (63) found that common disturbance in the discourse of patients was a lack of speech cohesion. However, compared to current studies, the method described in this study was superior in terms of investigating wide-ranging disturbances at a single point in time and at varying levels (53, 64). In addition, this method of discourse analysis was adaptable for analyzing speech in patients with schizophrenia. The speech data from this study concluded that the language impairment was caused by underlying thought disorder (63).

A study by Morice (65) differed to the study by Noel-Jorand, whereby the analyses of free speech samples were conducted on patients with schizophrenia, mania, and non-psychotic control. The language profiles contained syntactic variables reflecting the complexity, integrity, and fluency of spoken language. Linguistic differences between the three diagnostic groups enabled accurate (95%) classification by discriminant function analysis. Both studies suggest the important role for language analysis in psychiatric diagnosis (63, 65).

Moving away from a speech analysis approach, in a study by Rapp (66), the investigators used mental cognitive structures (objective taxonomies) and elements (case frames) in developing semantic networks that schematize speech through simulation methods by artificial intelligence (66). These computing-based neural networks (semantic networks) consisted of two main structures: case frames and object taxonomies (66–68). Node-based reasoning rules apply to object taxonomies (categories) and pathway-based reasoning rules apply to case frames. In this way, normal listeners might recognize speech as “crazy talk” based on violations of node- and pathway-based reasoning rules (66–68).

Older studies discussed in this section tended to study language and speech disturbances in patients with an established diagnosis of psychosis and/or affective disorder. These studies were disadvantaged by the lack of risk stratification tools such as the CAARMS (Criteria at Risk Mental State), which is widely used in many early intervention psychosis studies. However, current meta-analyses on CAARMS concluded that further research was required to improve its prediction of first episode of psychosis (69). On another note, old and current automated studies have not considered using a culturally sensitive interview instrument in assessing symptom profile in patients with first episode psychosis (70).

In conclusion, despite the different methodologies, these earlier studies demonstrated that speech and language disturbances can be quantified using computational and statistical methods. These studies also established that there was a recognizable pattern of disturbances in speech and language observed in those with a known diagnosis of mental illness that can be predicted with statistical accuracy.

### Semantic Methods: Review of Current Studies on LSA and Schizophrenia

In the following section, we will review current studies that have used NLP for analyzing semantic incoherence.

Machine learning has demonstrated an ability to detect subtle features of psychosis in language. Semantic coherence, semantic density, and acoustic analysis are the current methods used in detecting early signs of psychosis. Machine learning can measure the linguistic variables, semantic coherence and semantic density, and use of words relating to sound.

The use of computer-based assessment of natural language as a framework for measuring communication disturbances was described in an article by Cohen et al. This paper reviewed several studies that applied various computer-based assessments to natural language produced by adult patients with SMI. This review paper found that automated computerized methods were able to objectively evaluate patients in a reliable, valid, and efficient manner that complemented human ratings.

Bedi and Corcoran, in a proof-of-principle study in 2015, aimed to test automated speech analyses combined with machine learning to predict psychosis onset in youths at clinical high risk for psychosis. This study analyzed baseline interviews using automated analyses for semantic and syntactic features predicting later onset of psychosis. LSA was used to measure semantic coherence and two syntactic markers of speech complexity: maximum phrase length and use of determiners. These speech features predicted later development of psychosis with 100% accuracy and outperformed classification from clinical interviews. This method was found to have usefulness as an objective clinical test for psychiatry in measuring subtle clinically relevant mental state changes in emergent psychosis.

In a more recent study, Emery et al. used semantic density and latent content analysis to examine how people use words across sentences. This study found that compared to semantic coherence, semantic density was a better indicator of the mental processes that people used to form sentences. Low semantic density and talk about voices and sounds is a sign of conversion to psychosis. Low semantic density is also known as poverty of thought content, or vagueness. Those who later developed psychosis tend to use more sound-related words than the baseline and to use similar word meaning more frequently (71).

In the following section, we will review studies on acoustic analysis.

### Non-Semantic Methods: Review of Current Studies on Acoustic Analysis

Acoustic analysis is a computational method of analysis of the non-verbal aspects of speech features, non-semantic. Despite its existence for decades, application in research of SMI was modest. However, there is evidence that non-language neurocognitive abilities, for example, attention and acoustic measures of speech production and variability, were correlated with each other in patients with schizophrenia, schizotypal personality (a personality disorder characterized by paranoia and social anxiety, and by beliefs, behavior, and ways of speaking that are considered odd or eccentric), depression, and bipolar disorders (mental disorder marked by periods of elevation and depression and in extreme phases characterized by psychosis) (72, 73). The following key studies discussed in this section examine acoustic analysis as a computational technique of analysis of the non-verbal aspect of speech features and how its abnormalities in schizophrenia are measured.

Emotional expression is an essential function for daily life that can be severely affected in some psychological disorders. Laboratory-based procedures designed to measure prosodic expression from natural speech have shown early promise for measuring individual differences in emotional expression but have yet to produce robust within-group prosodic changes across various evocative conditions. Use of evocative slides organized according to either a dimensional (e.g., high and low arousal—pleasant, unpleasant, and neutral valence) or a categorical (e.g., fear, surprise, happiness) model produced robust changes in subjective state but only negligible change in prosodic expression. Alternatively, speech from the recall of autobiographical memories resulted in meaningful changes in both subjective state and prosodic expression (74).

Vocal expression is disrupted to some extent in psychiatric conditions. Cohen, in a study on vocal acoustic analysis as a biometric indicator of information processing, suggests that variability in vocal expression reflects availability of online resources (working memory and attention). This study aimed to establish the link between vocal expression and information processing in healthy adults. By using automated computerized analysis to measure vocal expressions, this study found that increased processing load resulted in longer pauses, fewer utterances, and greater overall silence and less variability in frequency and intensity levels. This study provided important information for the development of inexpensive automated non-invasive biometric measure of information processing (54).

Abnormalities in nonverbal communication are a hallmark of schizophrenia. Using statistical methods such as principal component analysis (PCA), Cohen et al. a) identified independent vocal expression measures from a large set of variables, b) quantified how patients with schizophrenia are abnormal with respect to these variables, c) evaluated the impact of demographic and contextual factors (e.g., study site, speaking task), and d) examined the relationship between clinically rated psychiatric symptoms and vocal variables. PCA identified seven independent markers of vocal expression. Most of these vocal variables varied considerably as a function of context and many were associated with demographic factors. After controlling for context and demographics, there were no meaningful differences in vocal expression between patients and controls. Within patients, vocal variables were associated with a range of psychiatric symptoms—though only pause length was significantly associated with clinically rated negative symptoms (75).

Measuring negative symptoms in schizophrenia is crucial for treating schizophrenia. A study by Cohen that used computerized analysis to measure patient speech showed that it was possible to measure flat affect, alogia, and anhedonia (positive emotion) using lexical–analytical software. These measures were examined in their relationship to clinically rated negative symptoms and social functioning, using natural speech samples that were analyzed for clinically rated flat affect and without flat affect. Computer-based inflection and speech rate measures were able to discriminate patients with flat affect from controls, and the computer-based measure of alogia and negative emotion significantly discriminated the flat and non-flat patients. Both the computer and clinical measures of positive emotion/anhedonia corresponded to functioning impairments (76).

In another study, Cohen improved the methodology in measuring emotional expression. Emotional expression in daily living is important and can be disrupted in many psychological disorders (e.g., schizophrenia). The present study examined the Computerized assessment of Affect from Natural Speech (CANS), a laboratory-based procedure that was designed to measure both lexical and prosodic expression from natural speech across a range of evocative conditions. Lexical and prosodic expression variables significantly changed across these conditions, providing support for using the CANS in further laboratory research (77).

In another study, Cohen compared degree of speech characteristics that were a reflection of psychiatric symptoms versus neurocognitive deficits. Diminished expressivity is a poorly understood, but important construct for a range of mental diseases. In the present study, Cohen used computerized acoustic analysis of natural speech to understand diminished expressivity in patients with schizophrenia and mood disorders. The method used was speech samples in response to a variety of laboratory stimuli and completed neuropsychological batteries assessing a range of abilities. For both the schizophrenia and mood disorder groups, attentional coding deficits were significantly correlated with increased pause time (at large effect size levels) and, for the schizophrenia group only, reduced prosody (also at a large effect size level). For the mood disorder but not the schizophrenia group, increased average pause time was also significantly associated with neurocognitive deficits on a range of other tests (medium to large effect size levels). Psychiatric symptoms were not significantly associated with speech characteristics for either group (generally, negligible effect sizes). These results suggest that there is a link between expressivity and neurocognitive dysfunctions for both patients with schizophrenia and mood disorders (78, 79).

Kessing (72) used voice analysis as an objective marker in bipolar disorder. This study found that changes in speech were sensitive and a valid measure of bipolar disorder. In this study, voice features were derived from behavioral smartphone data and electronic self-monitoring smartphone data. The statistical analysis used was based on random forest algorithm (72). The study found voice features as accurate, sensitive, and specific in the classification of manic or mixed state (72). Mota (80) also quantified difference in speech related to psychosis based on acoustics, which was recorded and represented as a graph. Compared to Kessing’s method of statistical analysis of different psychiatric disorders, the study by Mota used oral interviews as objective markers in making the differential diagnosis.

Reily (66) conducted acoustic and temporal analysis of speech in search for potential biomarkers for schizophrenia. This study had 39 patients with schizophrenia and 18 controls who were digitally recorded reading aloud an emotionally neutral text passage from a children’s story. From these recordings, temporal, energy, and vocal pitch features were automatically extracted. A classifier based on linear discriminant analysis (81) was used to differentiate between controls and patients with schizophrenia. The recordings showed that it was possible to differentiate patients with schizophrenia from control based on speech pause related parameters with 79.4% accuracy. The automated nature of this procedure also meant that near-perfect test–retest reliability can be expected for the same speech sample.

The studies we have discussed in this section used different methods of acoustic analysis, which indicates that measurement of incoherence is complex and cannot be reduced to a single method (66, 80). Conclusively, in these studies, automated acoustic analysis of speech was considered reliable and valid. Such findings have fueled theories that reductions in speech production and speech variability was a hallmark of neurocognitive dysfunction.

## Review of Current Studies Using Speech Graphs

Speech graph is another method of computational characterization of mental states. This method models co-occurring patterns between successive spoken words (82). The theoretical underpinning of this theory is based on mathematical structures used to model pairwise relations between objects. A graph is made up of vertices, nodes, or points connected by edges, arcs, and lines. The strength of this method is that it can be generalized to any language. The following studies describe the computational methods used to characterize abnormal mental states based on graph theory that were done in native Portuguese and translated to English.

In a study by Mota (83), speech dysfunction was measured using speech graph. Using complex network models, derived from graph theory, different aspects of non-pathological language were studied. A speech graph in this study represented a network with nodes connected by edges. In the case of language, nodes correspond to words and edges correspond to semantic and grammatical relationships. The interpretation of a graph’s meaning depended on what was being represented. However, the quantification of its structure sheds light on the relationship between language and altered mental states. In this study, the issue of classification accuracy was addressed by comparing the group classification obtained by the Node Base (NB) model with four other binary classifiers: Radial Basis Function (RBF) (83), Multi-Layer Perceptron (MLP) (83), Support Vector Machine (SVM) (83), and Decision Tree (DT) (83).

In another study by Mota (80), by analyzing the structural randomness of speech graph, connectedness was found to be decreased in schizophrenia. It provided a quantitative measurement of word salad as a fragmentation index (that tightly correlated with negative symptoms and predicted schizophrenia diagnosis during first clinical contact of recent-onset psychosis).

In a more recent study by Mota (84), the quantitative analysis of speech graphs complemented standard psychometric rating tools, as it resulted in an accurate sorting of schizophrenics and manics. Overall, the results point to automated psychiatric diagnosis based not on “what is said,” but on “how it is said.” Binary classifiers based on speech graph measures sorted schizophrenics from manics with up to 93.8% sensitivity and 93.7% specificity. In contrast, sorting based on the scores of two standard psychiatric scales (BPRS and PANSS) reached only 62.5% of sensitivity and specificity. The results demonstrate that alterations of the thought process manifested in the speech of psychotic patients can be objectively measured using graph-theoretical tools, developed to capture specific features of the normal and dysfunctional flow of thought, such as divergence and recurrence.

A limitation of the computational method in the studies by Mota et al. was that the issue of index severity was not factored into the classification (80, 83, 84).

Speech graphs have been shown to have high specificity and sensitivity for quantification of language dysfunction. However, despite this, the issue of dimensionality and taxonomy with machine learning algorithms remains a concern. Application of clustering algorithms to stratify psychiatric disorders poses problems as participants may not belong to any class. In addition, there is the question as to whether healthy participants should be clustered separately or included with other patients. Within the classification itself, some classes may be small or not well defined.

In the next few studies, we will briefly describe how Mota et al. linked age, level of education, and psychiatric state and subsequently used speech graph to analyze literary texts. By mapping out these links, Mota identified education as a cultural marker and drew correlation with psychopathology.

Discourse or spoken communication according to a study by Mota varies with age and level of education and psychiatric state. This study described the use of word graphs that have shown to provide behavioral markers of formal thought disorder in psychosis. This was achieved by tracking literacy acquisition in children with typical development. In this study, subjects with psychosis did not show dynamics in speech changes and present at adulthood a child-like discourse structure. Typical subjects increase the range of word recurrence over school years but the same feature in subjects with psychosis resists education (85).

Another study examined the relationship between memory and early school performances. Using graph theory, the link between the structure of children’s memories and their cognitive or academic performances was measured. Theory of mind correlated positively with word diversity and word-to-word connectivity and negatively with word recurrence (86).

A characteristic feature of psychosis is the prominence of loosened associative links in thought processes. Assessing this aspect of thought disorder is difficult as there are no markers that can reliably track the physiological effects of treatment in reducing thought disorders. A study by Palaniyappan et al. found that automated speech graph was able to reliably quantify structural speech disorganization. This study found that using structural and functional imaging, speech dysconnectivity and psychosis were due to neurodevelopmental deficits (87).

The above studies by Mota and others have demonstrated that discourse varies with age, education, psychiatric state, and time periods. However, the cultural dynamics of discourse structure remain to be quantitatively characterized. This study examined word graphs from verbal reports and literary texts spanning 5,000 years. In healthy subjects’ lexical diversity, graph size and long-range recurrence departed from initial near-random levels through a monotonic asymptotic increase across ages while short-range recurrence showed a corresponding decrease. These changes were explained by education and suggest a hierarchical development of discourse structure. In literature, monotonic asymptotic changes over time were remarkable. This study described Bronze Age texts as structurally similar to childish or psychotic discourses. However, in the period of cultural change, the subsequent texts show health adult pattern (84, 90).

To conclude, although speech graph methodology has been able to study discourse of different cultures through literary texts, more research is needed before generalizing this approach to indigenous cultures that are rich in oral and visual knowledge systems and mapping the link between indigenous education in pre-school, cognitive development, speech connectivity, and risk of developing psychosis.

## Discussion

The comprehensive review of current and past literature has highlighted the developments of advanced computer technology, specifically in the field of NLP and its application in psychiatric diagnoses. At a theoretical level, the results from these studies provide evidence for some of the hypotheses in schizophrenia and language dysfunction research. The cross-cultural studies have highlighted the importance of thought-implicit language and the connections between the systems underlying emotions and the various language and paralanguage processes.

Computational studies reviewed in this article were based on the concept that the computer system and method takes as an input a set of grammar and lexical items and generates an output that consists of semantic representations of disordered mental states. The methodology in these studies was conditioned by lexical and pragmatism rather than a language model that was based on structural factors as distinct from grammatical constructions based on the English language.

A key finding from these cross-cultural studies was the contentious subject of language relativity. These studies have highlighted the importance of thought-implicit language and the connections between the systems underlying emotions and various languages with space and time and how it shapes the manifestation of psychosis and schizophrenia. This leads to the next interesting finding in these cross-cultural studies of the role of culture itself. Culture in itself is a hierarchical semiotic system that consists of a set of functions correlated to linguistic codes used by social groups to maintain “coherence and cohesion.” This hierarchy system is based on subject, value system, and the world in which the subject is embedded. Thus, “coherence and cohesion” in this context differs significantly to the term “coherence and cohesion” derived from linguistic theory. The relevance of this perspective of indigenous communities could be further understood based on Kincaid’s convergence theory of communication, self-organization, and cultural evolution. Group-level boundaries are established by (and measured by) the flow of information through communication networks—both interpersonal and mass media linkages. The degree of cognitive, cultural convergence within a given group, organization, or society is determined by the extent to which its members share the same information over time (88). Hence, grammar of a particular language is not highly abstract or a static competence-driven system of knowledge. Instead, it is an emergent dynamic set of communicative discursive practices preferred in that linguistic community. Some of the discourse is highly grammaticalized and other parts are subject to varying degrees of conventionalization (88).

Western thinking distinguishes between the spoken word and emotions; Maori don’t draw such sharp distinction. Health is viewed as an interrelated phenomenon rather than an intrapersonal one. Healthy thinking is an integrative one, not analytical; explanations come from outside rather than inside. (89). Thus, concepts such as cognitive deficits and positive symptoms may take the form of an indigenous-based explanation that may not necessarily have a pathological basis.

These computational methods may well be a circuitous method in understanding the importance of expression of feelings and thoughts. Maori thinking and other indigenous cultures can be described as holistic. Understanding occurs less by division into smaller parts and the analytical approach than by synthesis into wider contextual systems so that any recognition of similarities is based on comparisons at a higher level of organization (89).

Another finding we felt was of importance was the need to extract psychological theory of cognition and consciousness from indigenous worldviews. This would form the basis of understanding how theories of cognition and consciousness in indigenous cultures relate to Western theorizing and research specifically in psychosis and schizophrenia research (the potential to modify and redefine existing Western concepts of cognition and linguistic theories).

As increasingly identifying psychosis and schizophrenia becomes less of a focus and more about meanings and outcomes and given that indigenous cultures hold different views about psychosis and spirituality with unclear boundaries, machine learning methods of screening will further assist with prediction and classification situations (92). In conclusion, it is evident that through critical discourse with indigenous communities, there is a need to develop indigenous research methods that can integrate social psychology, culture, and neuroscience and accommodate computational methods that can address at a computational level how psychosis and schizophrenia can be operationalized at an algorithmic level.

## Author Contributions

RR and HS conceived the presented review. RR and JK developed the theory and performed its articulation. HS and SP verified the analytical methods. JK encouraged RR to investigate the culture and psychiatric methods and HS and SP supervised the findings of this work. All authors discussed the results and contributed to the final manuscript.

## Conflict of Interest Statement

The authors declare that the research was conducted in the absence of any commercial or financial relationships that could be construed as a potential conflict of interest.
